# Supplementary material for: Early-life immune activation is a vulnerability factor for adult epileptogenesis in neurofibromatosis type 1 in male mice
Source: Front Neurol. 2024 Apr 15;15:1284574. doi: 10.3389/fneur.2024.1284574 (PMC11056566; doi:10.3389/fneur.2024.1284574)
Supplement: Supplementary file 2 [file Data_Sheet_2.docx]

***Supplementary Figures***


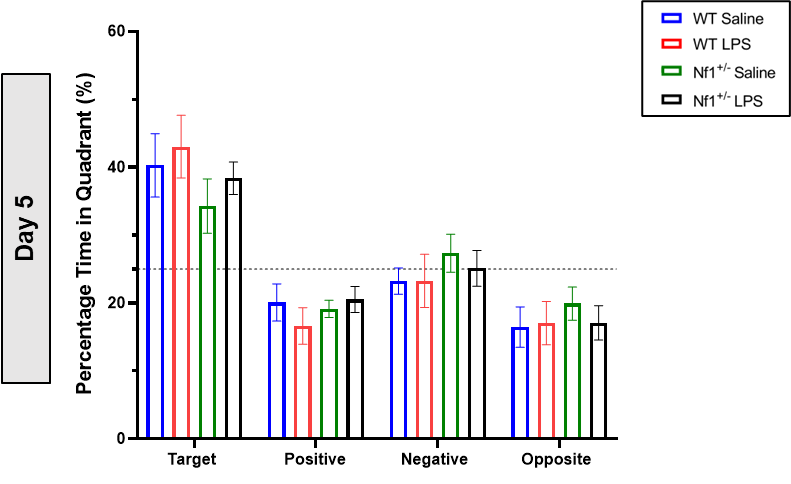


*** * ^#^ ***

**Supplementary Figure 1. Quadrant Exploration time in the Barnes Maze.** Bar graphs represent the average time spent in each of the four quadrants (as a percentage of the total time) during the probe trial on Day 5 of testing. The target quadrant contains the target hole. The dotted line represents chance. Data are reported as mean ± SEM. Two-Way repeated measures ANOVA followed by Dunnett’s post-hoc for within-group differences, Tukey’s post-hoc for between-group differences. Control WT and *Nf1^+/-^* mice exhibited target quadrant preference during the probe trial: Control WT mice explored the Target quadrant significantly more than any other quadrant in the maze [Dunnett’s post hoc (*vs.* Target), Positive: *p* < 0.001; Negative: *p* = 0.002; Opposite: *p* < 0.001] and control *Nf1^+/-^* mice showed bias towards the Target quadrant as well [(*vs.* Target), Positive: *p* = 0.004; Opposite: *p* = 0.007] although this was not generalized to all non-target quadrants [(*vs.* Target), Negative: *p* = 0.306]. However, there were no differences in the percentage of time spent exploring the Target quadrant when comparing between control WT and *Nf1^+/-^* mice. Similarly, both LPS-challenged WT and LPS-challenged *Nf1^+/-^* mice displayed target quadrant discrimination, as evidenced by exploring the Target quadrant significantly more than any other quadrant [WT (*vs.* Target), Positive: *p* < 0.001; Negative: *p* = 0.001; Opposite: *p* < 0.001, *Nf1^+/-^* (*vs.* Target), Positive: *p* = 0.004; Negative: *p* = 0.041; Opposite: *p* < 0.001]. The percentage of time spent exploring the Target quadrant was also not statistically different when comparing LPS-challenged WT and LPS-challenged *Nf1^+/-^* mice, and similarly not statistically different when compared to their saline-matched controls. **p*<0.05 compared to all other quadrants for that group, #*p*<0.05 compared to positive and opposite quadrants for that group.


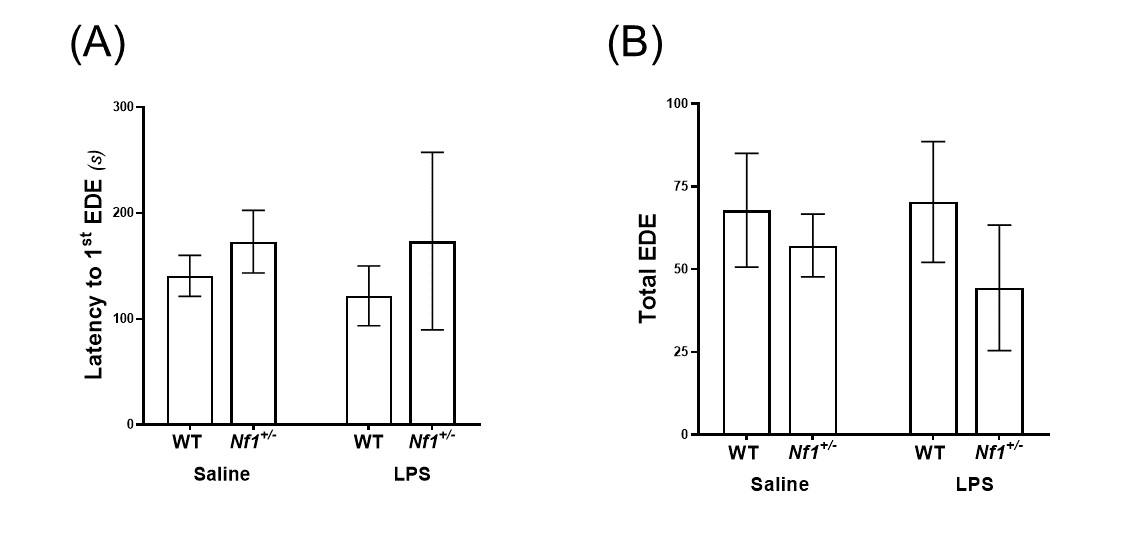


**Supplementary Figure 2. The latency to the first epileptiform discharge event (EDE) and total EDE count in *Nf1^+/-^* and WT adult mice exposed to LPS or saline as neonates**. Bar graphs represent the Mean ± SEM, and data were analyzed in a One-Way ANOVA. There were no statistical differences found in the latency to the first EDE nor in the total EDE count across all the groups.


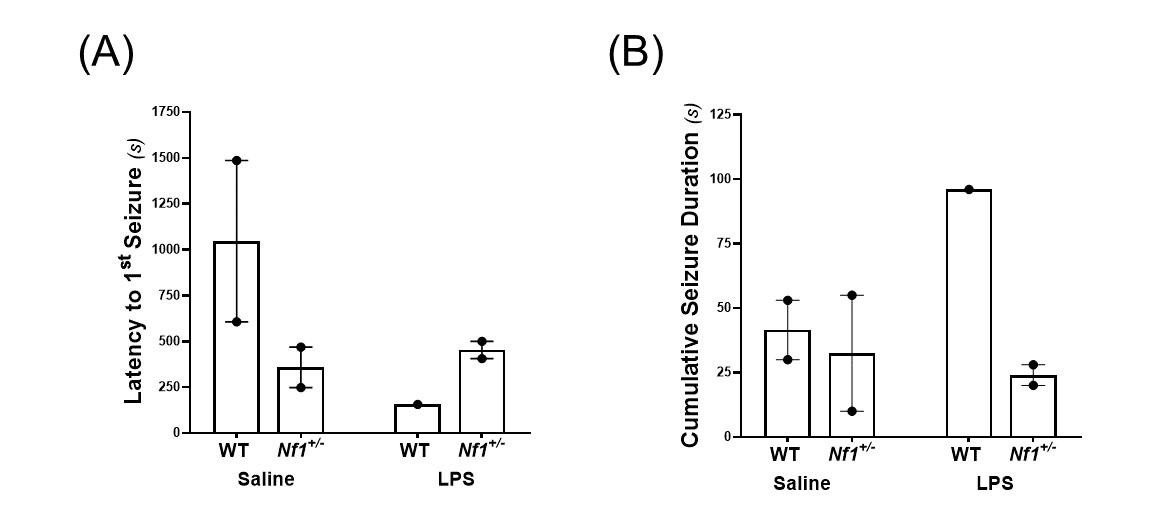


**Supplementary Figure 3. The latency to the first PTZ-induced seizure and cumulative seizure duration in *Nf1^+/-^* and WT adult mice exposed to LPS or saline as neonates**. An individual data point represents one mouse and, whenever appropriate, bar graphs displayed the mean ± SEM. PTZ-induced seizures were detected 3/8 control WTs, 2/6 control *Nf1^+/-^* mice, 1/8 LPS-challenged WTs and 3/6 LPS-challenged *Nf1^+/-^* mice with no statistical differences in the proportion of mice that developed PTZ-induced seizures (χ2 (3, N = 28) = 1.11; p = 0.774). The average latency to the first PTZ-induced seizure in control *Nf1^+/-^* mice appears to be shorter than that observed in control WT mice. The cumulative seizure durations, however, appear comparable across these control groups. Compared to their saline controls, LPS-challenged *Nf1^+/-^* mice appeared to have a comparable seizure latency and cumulative seizure duration. Only one LPS -challenged WT mouse developed PTZ-induced seizures and compared to the saline controls, appeared to have a shorter seizure latency and a longer cumulative seizure duration. However, statistical comparisons of these seizure latencies and cumulative seizure durations were not possible due to the small number of mice that developed PTZ-seizures.
